# Prospective, historically controlled study to evaluate the efficacy and safety of a new paediatric formulation of nifurtimox in children aged 0 to 17 years with Chagas disease one year after treatment (CHICO)

**DOI:** 10.1371/journal.pntd.0008912

**Published:** 2021-01-07

**Authors:** Jaime Altcheh, Luis Castro, Juan C. Dib, Ulrike Grossmann, Erya Huang, Guillermo Moscatelli, Jimy José Pinto Rocha, Teresa Estela Ramírez, on behalf of the CHICO Study Group

**Affiliations:** 1 Hospital de Niños Ricardo Gutiérrez and Instituto Multidisciplinario de Investigacion en Patologias Pediatricas (IMIPP), CONICET-GCBA, Buenos Aires, Argentina; 2 Centro de Atencion e Investigacion Medica S.A. (CAIMED–Yopal), Yopal, Colombia; 3 Universidad del Norte, Barranquilla, Colombia; 4 Bayer AG, Research and Development Pharmaceuticals, Berlin, Germany; 5 Bayer US LLC, Whippany, New Jersey, United States of America; 6 Fundación CEADES–Plataforma de Atención Integral a los Pacientes con Enfermedades de Chagas, Cochabamba, Bolivia; 7 Centro de Enfermedad de Chagas y Patologias Regionales, Santiago del Estero, Argentina; Universidad de Buenos Aires, ARGENTINA

## Abstract

Nifurtimox is a recommended treatment for Chagas disease, but data from treated children are limited. We investigated the efficacy, safety and tolerability of nifurtimox administered as divisible, dispersible 30 mg and 120 mg tablets in children with Chagas disease. In this blinded, controlled study conducted January 2016–July 2018, 330 patients aged <18 years from 25 medical centres across three South American countries were randomised 2:1 to nifurtimox 10–20 mg/kg/day (aged <12 years) or 8–10 mg/kg/day (aged ≥12 years) for 60 days (n = 219), or for 30 days plus placebo for 30 days (n = 111) (ClinicalTrials.gov NCT02625974). The primary outcome was anti-*Trypanosoma cruzi* serological response (negative seroconversion or seroreduction ≥20% in mean optical density from baseline determined by two conventional enzyme-linked immunosorbent assays) at 12 months in the 60-day treatment group versus historical placebo controls. Nifurtimox for 60 days achieved negative seroconversion (n = 10) and seroreduction (n = 62) in 72 patients (serological response 32.9%; 95% confidence interval [CI] 26.4%, 39.3%, of all treated patients), confirming superiority relative to the upper 95% CI of 16% for controls. In patients aged <8 months, 10/12 treated for 60 days (83.3%) and 5/7 treated for 30 days (71.4%) achieved negative seroconversion. Overall serological response was lower for 30-day than for 60-day nifurtimox (between-treatment difference 14.0% [95% CI 3.7%, 24.2%]). The frequency of *T*. *cruzi-*positive quantitative polymerase chain reactions decreased substantially from baseline levels (60-day regimen 53.4% versus 1.4%; 30-day regimen 51.4% versus 4.5%). Study drug-related treatment-emergent adverse events (TEAEs), which were observed in 62 patients (28.3%) treated for 60 days and 29 patients (26.1%) treated for 30 days, were generally mild or moderate and resolved without sequelae; 4.2% of all TEAEs led to nifurtimox discontinuation. Age- and weight-adjusted nifurtimox for 60 days achieved a serological response at 12 months post-treatment that was superior to historical placebo, was well tolerated and had a favourable safety profile in children with Chagas disease. Although, at 1 year serological follow-up, efficacy of the shorter nifurtimox treatment was not comparable to the 60-day treatment regimen for the overall study population, further long-term follow-up of the patients will provide important information about the progress of serological conversion in children treated with nifurtimox, as well as the potential efficacy difference between the two regimens over time.

## Author summary

New cases of Chagas disease, which is caused by infection with the protozoan parasite *Trypanosoma cruzi*, mostly occur in children. Undiagnosed, or without effective treatment following diagnosis, the infection will persist. Around a third of those with chronic Chagas disease will develop serious disorders, such as heart or digestive system disease. Thus, early effective treatment is important to eliminate the parasite. In this prospective study, we used divisible, dispersible formulations of nifurtimox in 30 mg and 120 mg tablets to treat patients aged from newborn to 17 years with age- and weight-adjusted doses for 60 days or for 30 days plus placebo for 30 days. After 12 months, we found serological response rates of 33% and 19% of children in the 60-day and 30-day treatment groups, respectively. The proportion of patients with parasite detectable in blood by polymerase chain reaction tests also decreased substantially with both treatments during this period. Nifurtimox was safe and well tolerated in paediatric patients. The study continues and will provide long-term data from follow-up of 4 years after end of nifurtimox treatment.

## Introduction

Chagas disease, which is caused by infection with the protozoan parasite *Trypanosoma cruzi*, poses a substantial and evolving global burden in terms of morbidity, mortality and costs [1]. The World Health Organization (WHO) estimates that over 10,000 people die from the manifestations of Chagas disease annually and about 8 million individuals are infected worldwide, mostly in Latin America [2]. The burden of morbidity associated with Chagas disease is greater than that of any other parasitic disease, with mortality among infected infants of 5–20% [3].

Following infection with the parasite, and if left untreated, Chagas disease progresses through an incubation period to an initial acute phase of 6–8 weeks, followed by a chronic phase, which can be determinate or indeterminate, lasting years or decades [4,5]. Between 70% and 80% of patients display the indeterminate chronic form without clinical signs or symptoms, and therefore infection can only be confirmed by serological testing [4,5]. In up to 40% of patients, Chagas disease can progress to the determinate or symptomatic chronic form 10–30 years after the initial infection, with affected patients suffering a range of clinical manifestations including cardiac, digestive system, neurological, or combined disorders [4–7].

A variety of methods are used to diagnose Chagas disease depending on the phase of the disease [5]. During the acute phase, diagnosis is made by direct parasitological tests of fresh blood samples and by molecular methods such as polymerase chain reaction (PCR). In the indeterminate chronic phase, serological methods are used to demonstrate the presence of specific anti-*T*. *cruzi* antibodies. Current guidelines advise using at least two different serological methods for diagnosis [8].

*Trypanosoma cruzi* infection is curable if treatment is initiated soon after infection, and antiparasitic treatment is likely to prevent or curb disease progression in the chronic phase [9]. Accordingly, current guidelines recommend treatment with trypanocidal drugs in all patients with acute phase disease or congenital infection, those with immunosuppression or at risk of reactivated infection, and many patients with chronic infection, such as those in the indeterminate phase of the disease or with minimal cardiac involvement, and women of childbearing age (to avoid transplacental transmission) [2,10,11]. Notably, treatment with trypanocidal drugs is recommended by the Pan American Health Organization for all children younger than 18 years in the Americas, including newborns of infected mothers who have positive parasitology for *T*. *cruzi*, and women of childbearing age to prevent transplacental transmission [12].

Currently, benznidazole and nifurtimox are the only antitrypanosomal agents approved for Chagas disease. Although both drugs act through the formation of cytotoxic free radical intermediates and electrophilic metabolites generated by nitroreductases, enzymatic activation of nifurtimox also forms superoxide anion and hydrogen peroxide that cause breakdown of parasite deoxyribonucleic acid (DNA) by creating oxidative stress [5,13]. In individuals with acute Chagas disease or early congenital disease, both agents have been found to shorten the course of the illness and reduce both symptom severity and duration of parasitaemia, leading to cure rates of up to 100% in neonates and of 80–90% in children treated early in the disease course. However, treatment effectiveness appears to be lower during the chronic phase and it is acknowledged that the earlier children are treated, the higher the rate of disappearance of *T*. *cruzi* antibodies (negative seroconversion) [4,5]. The occurrence of treatment-related adverse effects is common, however, and historically has contributed to treatment discontinuation in 20–25% of patients [9]. Recent studies have investigated alternative regimens, such as shorter duration and/or lower doses, to improve treatment tolerability and adherence.

Nifurtimox is classified by the WHO as a vitally important medicine and is on their Model List of Essential Medicines [14]. Nifurtimox is licensed for use in Argentina, Chile, El Salvador, Guatemala, Honduras and Uruguay, but is available in the USA under an investigational protocol from the Centers for Disease Control and Prevention (CDC) [11]. Previous studies have demonstrated the efficacy and tolerability of nifurtimox in the treatment of patients, including some infants and children, with Chagas disease [15–17]. Currently, only nifurtimox 120 mg tablets are available, so a paediatric formulation is needed to facilitate administration and improve dosing accuracy in children. Our study was undertaken to increase understanding of the effects and outcomes of nifurtimox treatment in children with Chagas disease, using divisible and dispersible tablets of two different dose strengths, 30 mg and 120 mg [18], administered in a modified age- and weight-adjusted treatment regimen for 60 days and for half this duration.

## Methods

### Ethics statement

The study was approved by the respective independent ethics committees of participating investigational sites (Argentina: Comité de Ética, Hospital General de Agudos J. A. Fernández, Buenos Aires; Comité de Ética Independiente en Investigación Clínica "Dr. Carlos A. Barclay", Buenos Aires; Comité Institucional de Revisión de Protocolos de Investigación, Hospital de Niños Sor María Ludovica, La Plata; Comité de Ética en Investigación del Hospital de Niños Ricardo Gutiérrez, Buenos Aires; Comité de Ética en Investigación, San Miguel de Tucumán; Comité de Ética en Investigación del Hospital General de Niños, Buenos Aires; Comité de Ética en Investigación Hospital FJ Muñiz, Buenos Aires; Comité Independiente de Ética Médica del Noroeste Argentino, San Miguel de Tucumán; Comité de Ética del Hospital Pediátrico Dr Humberto Notti, Mendoza; Comité de Bioética, Hospital Dr Fernando Barreyro, Posadas. Colombia: Comité de Ética en Investigaciones de la Fundación Cardiovascular de Colombia, Floridablanca; Centro de Atención e Investigación Médica CAIMED S.A., Bogota; Comité de Ética en Investigación Hospital Mental Antioquia, Bello; Universidad del Norte, Barranquilla; Bolivia: Comité de Ética de CEADES, Cochabamba; Comité de Ética Hospital Manuel Ascencio Villarroel, Punata). Written informed consent of the patient and/or their parent(s) or legally authorised representative(s) was obtained prior to screening according to age and local regulations. In addition, depending on their age, patient assent was obtained if required by locally applicable laws and regulations in each country.

### Study design

This was a phase 3, randomised, quadruple-blind (see below), parallel-group, historically controlled, comparative superiority study in children with Chagas disease (ClinicalTrials.gov NCT02625974). The primary objective of the study was to assess the superiority of nifurtimox treatment for 60 days to historical untreated control at 12-month follow-up by seroreduction compared with baseline in patients aged ≥8 months to 17 years or negative seroconversion in all patients.

We used an external, historical-control design referring to data from two placebo-controlled trials of benznidazole conducted in children with Chagas disease [19,20]. One trial studied 129 symptom-free children aged 7–12 years with positive Chagas disease serology from a rural area of Brazil [20], the other studied 106 children aged 6–12 years mostly from a rural area of Argentina [19]; exclusion criteria in the latter study included chronic health conditions or the presence of any acute infectious disease [19]. Both studies saw 5% seroconversion rates (based on conventional ELISA serology) in their placebo groups, indicating some consistency of response between the two cohorts. The higher upper interval of the associated 95% CI values (16%) [19] was stipulated as the threshold for superiority over placebo in our study. The study was also designed to compare the effectiveness of nifurtimox treatment for 60 days and 30 days at 12-month follow-up by seroreduction and negative seroconversion.

The study was conducted between January 2016 and July 2018 at 25 hospital sites and treatment centres across South America (Argentina, n = 18, Bolivia n = 3, Colombia n = 4; see S1 Text in the online supplement). The design and all aspects of the conduct, evaluation and documentation of the study conformed with good clinical practice guidelines and the guiding principles of the current version of the Declaration of Helsinki. The study also complied with applicable local law(s) and regulation(s), and all information identifying patients was collected, stored and analysed in strict confidence and in accordance with local data protection laws.

### Study participants

The study included boys and girls aged from newborn to less than 18 years with a diagnosis of Chagas disease. Diagnosis was confirmed in patients aged <8 months at randomisation by direct observation of *T*. *cruzi* using a parasitological concentration test performed according to standard of care in each local laboratory. For patients aged between 8 months and less than 18 years, diagnosis was confirmed by positive results from both recombinant and total purified antigen conventional enzyme-linked immunosorbent assay (ELISA) tests (conducted by the local laboratory). Infants aged <28 days born preterm (gestational age <37 weeks), or weighing <2.5 kg at birth or with a maximum Apgar score <7 at 5 minutes after birth were excluded, as were patients with known Chagas-related cardiac, gastrointestinal or neurological disease documented in their medical history. Full exclusion criteria are provided in S2 Text in the online supplement. If sexually active, females who had experienced menarche and male patients were required to agree to use adequate contraception from the time of giving informed consent/assent until 3 months after the last study drug administration.

### Randomisation and masking

Patients were randomised 2:1 to nifurtimox 60-day or 30-day treatment regimens (see *Procedures*). A random allocation sequence was generated by a separate panel, independent of the study team, and implemented via an Interactive Voice Response System or Interactive Web Response System [21], according to investigator preference. To help maintain blinding, placebo tablets were colour-matched to nifurtimox tablets. All patients, care providers, research personnel (including the investigators and other study site staff) and outcomes assessors were blinded to treatment assignment. Using age at randomisation, patients were also assigned to the following four age-related strata, based on previous studies and age-based approaches to disease diagnosis: 0 to 27 days, 28 days to less than 8 months, 8 months to less than 2 years, and 2 years to less than 18 years.

### Procedures

**Fig 1:**
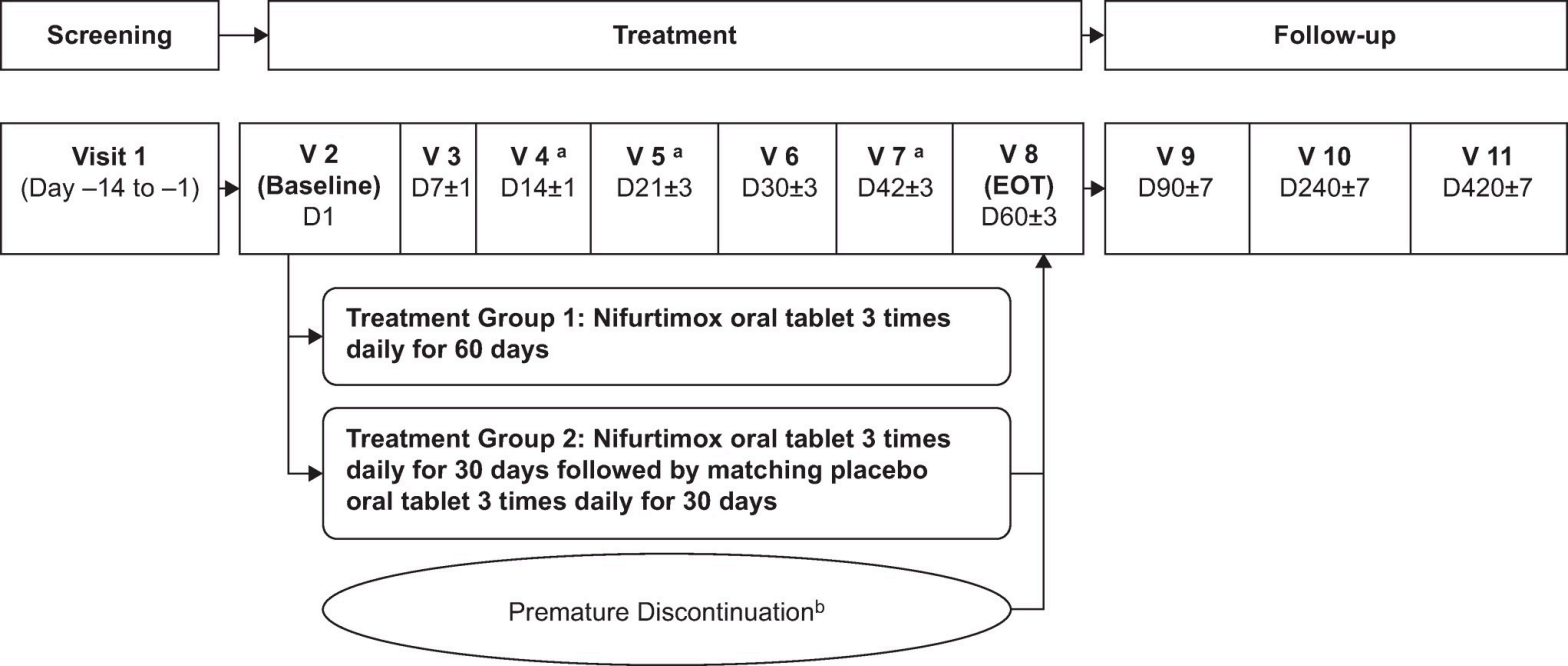
Schedule of study visits during treatment and follow-up. D, day; EOT, end of treatment; V, visit. ^a^ At Visits 4, 5 and 7, the occurrence of adverse events, the use of concomitant medications, and treatment compliance were assessed via telephone. ^b^ Patients who discontinued treatment prematurely returned to the investigating centre for assessments at Visits 3, 6 and 8 (EOT), and underwent telephone assessments as described for Visits 4, 5 and 7. If patients were unable/unwilling to do so, they had to return to the investigational site 30 (±3) days after the last dose of study drug for EOT (Visit 8) assessments, and undergo telephone assessments as described for Visits 4, 5 and 7. If the patients was unable/unwilling to return to the clinic for the EOT Visit, then a telephone assessment as described for Visits 4, 5 and 7 was performed in lieu of Visit 8 assessments.

Patients were randomised to nifurtimox for 60 days (60-day treatment regimen) or nifurtimox for 30 days followed by matching placebo for 30 days (30-day treatment regimen) (Fig 1). Nifurtimox 30 mg and 120 mg tablets (Bayer HealthCare AG, Germany) were administered at 10–20 mg/kg/day for patients <12 years of age and of body weight <40 kg, and 8–10 mg/kg/day for patients ≥12 years of age and of body weight ≥40 kg, in three divided doses with food. This age- and weight-adjusted dosing was designed to achieve comparable nifurtimox exposure across the paediatric age range.

Both 30 mg and 120 mg tablets were scored to enable division into equal halves of nifurtimox 15 mg or 60 mg, respectively. The tablets were formulated for rapid disintegration to allow administration to patients unable to swallow whole tablets. For these patients, the tablet was dissolved in approximately 5 mL water and taken immediately with food.

The safety and efficacy of study treatment were assessed at regular investigational site visits (Fig 1). For efficacy, blood samples were obtained for conventional ELISA tests, for non-conventional serological testing using the *T*. *cruzi* F-29 flagellar protein, and for detection of *T*. *cruzi* DNA using quantitative PCR (qPCR) immediately before treatment (baseline) and on Days 7 (±1), 30 (±3), 60 (±3), 240 (±7) and 420 (±7) after the start of treatment. *Trypanosoma cruzi* parasitological concentration tests (in children <8 months of age at randomisation only) were performed before treatment and on Days 7 (±1), 30 (±3), 60 (±3) and 90 (±7) after the start of treatment. Indirect haemagglutination (IHA) was carried out on samples taken before and on Day 420 (±7) after the start of treatment. Commercially available assay kits were used for ELISA (Chagatest ELISA recombinante v3.0 and Chagatest ELISA lisado; Wiener lab, Rosario, Argentina) and IHA (Chagatest HAI; Wiener lab) tests. For ELISA tests, optical density was measured at 450/620–650 nm using a bichromatic microplate reader. The qPCR tests amplified the *T*. *cruzi* satellite DNA region.

Compliance with study drug treatment was assessed at Visits 3–7 by pill count and by a daily diary. The diary was completed by the patients or their parents or carers and was reviewed and documented. For pill count, patients or their carers were provided with nifurtimox tablets together with detailed dosing information at specific visits by study staff; the total number of tablets provided exceeded that required in each case. At the next study visit, a pill count was performed and compliance evaluated and categorised according to the actual number of tablets used compared with that recommended into three groups: ≤80%, >80–120%, and >120%. Signs and symptoms of Chagas disease were assessed during treatment and follow-up.

### Outcomes

The primary outcome was serological response to treatment assessed as seroreduction (in patients aged between 8 months and <18 years at randomisation) or negative seroconversion (in all patients) as the measure of efficacy of the 60-day nifurtimox regimen at 12-month follow-up (360 days after the end of treatment) compared with historical placebo controls. Seroconversion was defined as a negative anti-*T*. *cruzi* immunoglobulin G concentration by two conventional ELISA tests (total purified antigen ELISA and recombinant ELISA). Seroreduction was defined as a ≥20% reduction in mean optical density measured in the two conventional ELISA tests compared with baseline. To define this reduction threshold, we used the published results of a previous placebo-controlled clinical trial in children aged 6–12 years with early chronic phase Chagas disease who were treated with benznidazole 5 mg/kg for 60 days and followed for 48 months [19]. At 12 months, the reduction in mean disease-specific antibody concentration measured as ELISA optical density was 21% compared with baseline. This reduction was similar to that found in a controlled trial in children aged 6–12 years with early chronic Chagas disease treated with 7.5 mg/kg benznidazole for 60 days, which also showed a reduction in antibody concentration of approximately 20% at 12 months [20]. Significant reductions in anti-*T*. *cruzi* antibody concentration (i.e., seroreduction) measured by conventional serology after antitrypanosomal treatment seem to be predictive of subsequent negative seroconversion [22–24].

Secondary outcomes and further exploratory objectives were: treatment response at 12-month follow-up for 30-day versus 60-day treatment; evaluation of IHA and F-29 serology; comparability of the 30-day versus 60-day nifurtimox regimens using qPCR at 12-month follow-up; and safety of nifurtimox. The 30-day regimen was included to investigate the effectiveness of exposure to nifurtimox for a period shorter than 60 days.

For safety, treatment-emergent adverse events (TEAEs) were assessed at each visit (Days 1, 7 (±1), 14 (±1), 21 (±3), 30 (±3), 42 (±3), 60 (±3), 90 (±7), 240 (±7) and 420 (±7)) and classified as mild (usually transient, requiring minimal intervention and not interfering with activities of daily living [ADL]), moderate (alleviated by specific intervention, interfering with ADL and causing discomfort but posing no significant risk or permanent harm) or severe (requiring intensive intervention, possibly hospitalisation, interrupting ADL and posing a significant risk of harm). Patients also underwent a physical examination, including measurement of body weight, height/length and vital signs, and a neurological examination; intake of concomitant medication was also recorded. At site visits on Days 1, 7 (±1), 30 (±3), 60 (±3) and 90 (±7), a 12-lead electrocardiogram (ECG) was obtained and pregnancy testing was carried out for females of childbearing age. Blood samples for haematological, coagulation and blood chemistry evaluation, and urine samples for urinalysis were collected on Days 7 (±1), 30 (±3), 60 (±3) and 90 (±7).

### Statistical analyses

The primary variable used as a measure of treatment response was negative seroconversion or ≥20% seroreduction at 12 months post-treatment using the two conventional ELISA serology tests. For the primary efficacy analysis, the difference in the proportion of nifurtimox-treated patients with negative seroconversion or seroreduction (60-day regimen) and the proportion estimated from historical data was tested using an asymptotic 2-sided 95% confidence interval (CI) with a continuity correction of 1/2n for a single proportion. For the primary objective, superiority over historical placebo control was confirmed if the lower limit of the 95% CI for nifurtimox treatment response (60-day regimen) at 12 months was greater than 16%, which is the larger of the upper 95% CI limits derived from two published studies (both found a 5% clinical response rate for the placebo group at 3-year [95% CI 1%, 13%] [19] and 4-year [95% CI 1%, 16%] [18] follow-up).

In a secondary analysis of the primary efficacy variable, the proportions of patients with negative seroconversion or seroreduction in the 60-day and 30-day nifurtimox regimens were compared using an asymptotic 2-sided 95% CI for the difference of two independent proportions. A between-treatment group difference in response rate for the 60-day and 30-day nifurtimox regimens was confirmed if the 95% CI did not span zero.

The efficacy and safety analyses used the full analysis set (FAS), which included all patients who, according to their compliance record, received at least one dose of study drug, irrespective of the dose regimen assigned. Analyses were also conducted using the per-protocol set, which included patients treated with study drug who had no major protocol deviations. With a sample size of 260 for the 60-day regimen the power was 99% for the lower limit of the 95% CI to be greater than 16%. The number of patients in the 30-day treatment regimen was based on the width of a 95% CI for the difference of two proportions. A sample size of 130 (30-day) together with 260 (60-day) will produce a CI with a half-width of approximately 20%. All significance tests were conducted using a 2-sided alpha level of 0.05, and CIs were 2-sided 95% intervals.

All analyses of safety variables were descriptive, and no formal testing was performed. No imputations were made for missing values occurring in the safety and background variables. All statistical analyses were performed using Statistical Analysis System version 9.2 or higher (SAS institute, Cary, NC, USA).

## Results

### Patient characteristics

**Fig 2:**
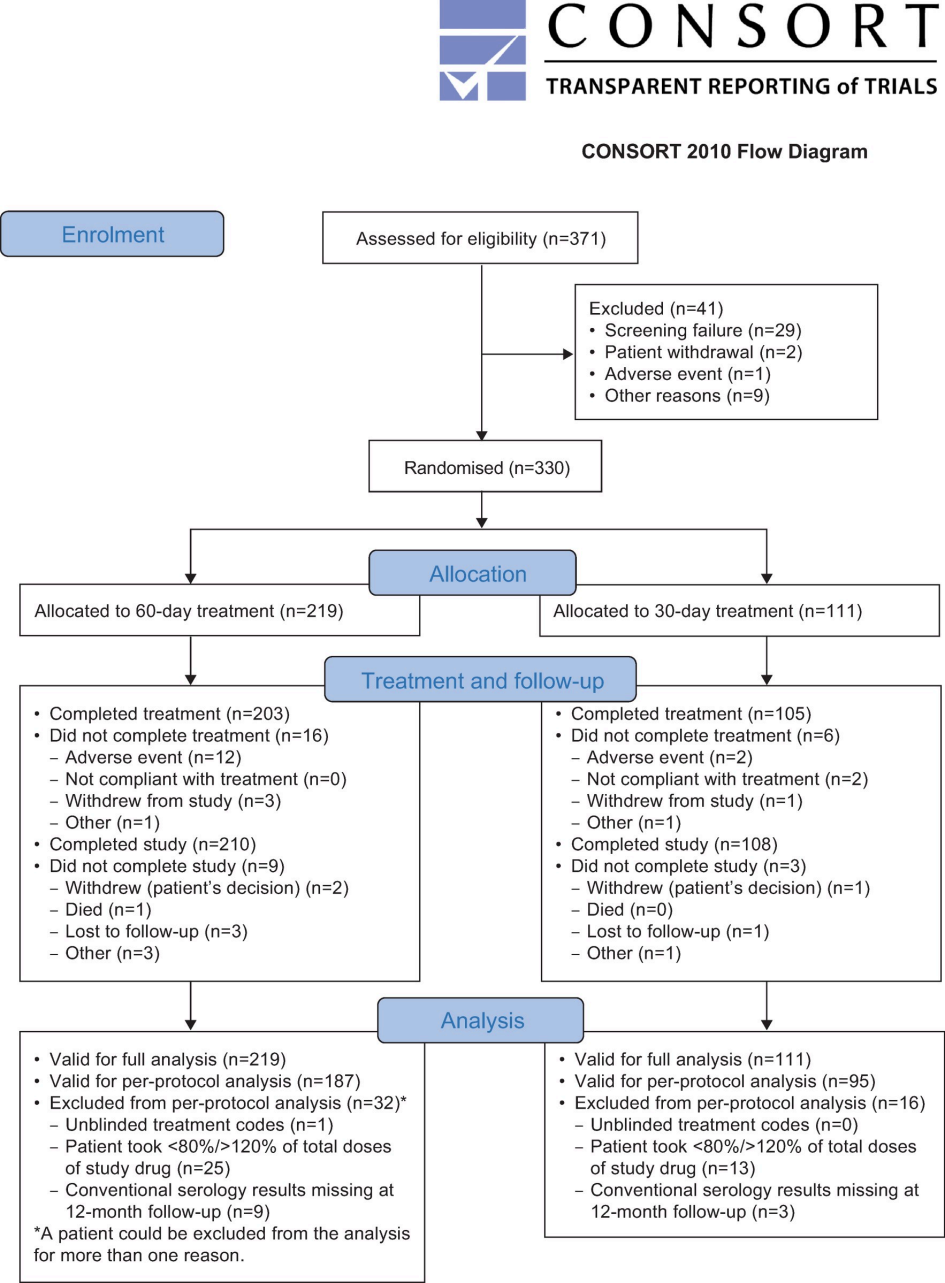
Patient disposition.

Between January 2016 and July 2018, 371 patients were enrolled in the study, 330 of whom were randomly assigned to nifurtimox treatment for 60 days (n = 219) or 30 days (n = 111) (Fig 2). Slightly more females than males were included (53.9% versus 46.1%, respectively) and most patients (86.7%) were aged 2–<18 years (Table 1). Pathogen-specific testing at screening by local laboratories provided a positive diagnosis of Chagas disease in all patients. Tests at a central study laboratory showed a negative result in one patient for each ELISA test; however, local laboratory results were positive for both patients, confirming the diagnosis of Chagas disease in all 330 patients in the FAS population (Table 2). Based on the proportion of planned and actual number of tablets taken, mean (standard deviation [SD]) treatment compliance was 92.3% (12.8%) in the nifurtimox 60-day treatment group and 92.9% (14.0%) in the 30-day treatment group (S1 Table).

**Table 1:** Patient characteristics.

	Nifurtimox 60-day regimen (n = 219)	Nifurtimox 30-day regimen (n = 111)	Total (N = 330)
Sex, n (%)			
Male	100 (45.7)	52 (46.8)	152 (46.1)
Female	119 (54.3)	59 (53.2)	178 (53.9)
Age group, n (%)			
0 to 27 d	4 (1.8)	3 (2.7)	7 (2.1)
28 d to <8 m	8 (3.7)	4 (3.6)	12 (3.6)
8 m to <2 y	17 (7.8)	8 (7.2)	25 (7.6)
2 y to <18 y	190 (86.8)	96 (86.5)	286 (86.7)
Weight, median (range), kg	31.5 (2.9–95.0)	39.0 (2.5–72.8)	34.0 (2.5–95.0)
Height, median (range), cm	136 (48–177)	143 (47–171)	139 (47–177)
BMI, median (range), kg/m^2^	17.85 (10.3–32.1)	18.63 (10.8–30.6)	18.20 (10.3–32.1)

d, days; m, months; y, years; BMI, body mass index.

**Table 2:** Serological and parasitological test results at baseline (full analysis set).

	Nifurtimox 60-day regimen (n = 219)	Nifurtimox 30-day regimen (n = 111)	Total (N = 330)
Total purified antigen ELISA test			
Reactive, n (%)	219 (100)	110 (99.1)	329 (99.7)
Non-reactive, n (%)	0	1 (0.9)	1 (0.3)*
OD values, mean (SD)	1.474 (0.553)	1.532 (0.533)	1.494 (0.546)
Recombinant ELISA test*			
Reactive, n (%)	219 (100)	110 (99.1)	329 (99.7)
Non-reactive, n (%)	0	1 (0.9)	1 (0.3)*
OD values, mean (SD)	2.735 (0.64)	2.765 (0.605)	2.745 (0.628)
Quantitative PCR test, n (%)			
Detectable	117 (53.4)	57 (51.4)	174 (52.7)
Non-detectable	99 (45.2)	53 (47.7)	152 (46.1)
Non-evaluable	1 (0.5)	1 (0.9)	2 (0.6)
Missing	2 (0.9)	0	2 (0.6)
Direct parasitological concentration test for *T*. *cruzi*^†^, n (%)			
Positive	12 (5.5)	7 (6.3)	19 (5.8)
Negative	0	0	0

ELISA, enzyme-linked immunosorbent assay; OD, optical density; SD, standard deviation; PCR, polymerase chain reaction.

*A non-reactive, i.e. negative, result was recorded for different single patients for each ELISA test. However, local laboratory results used for assessment of patient’s eligibility were positive for both patients, confirming the diagnosis of Chagas disease in all 330 patients in the FAS population.

^†^Patients aged <8 months (Note: the denominator for the percentage in brackets is the total number of patients in the respective treatment group; however, positive tests results were observed in all patients <8 months of age).

### Primary outcome

Treatment with nifurtimox for 60 days achieved negative seroconversion in 10 patients and seroreduction in 62 patients (Table 3), equating to a serological response to treatment of 32.9% (95% CI 26.4%, 39.3%). The lower 95% CI limit was greater than 16%, confirming the superiority of the 60-day nifurtimox regimen over historical placebo. The treatment response rate for the 30-day nifurtimox regimen was 18.9% (Table 3), yielding a 14.0% difference between the rates for the two regimens, with an asymptotic 2-sided 95% CI (3.7%, 24.2%) confirming a difference in efficacy between the two treatment regimens. Considering differences in treatment response by age group, the 60-day regimen was also more effective than the 30-day regimen among children aged 2–<18 years (difference 14.9% [95% CI 5.4%, 24.3%]). In patients younger than 8 months, negative seroconversion was observed in 10/12 patients (serological response 83.3%) who received nifurtimox for 60 days and 5/7 patients who were treated for 30 days (serological response 71.4%) (Table 3). In children younger than 2 years, treatment response rates of the two regimens appeared to be similar; however, no formal comparative test was performed (Table 3). The results for patients with no missing data or other major protocol deviation (per-protocol analysis set) were consistent with the results of the full analysis set.

**Table 3:** Serological responses to 60-day and 30-day nifurtimox (NFX) treatment assessed by conventional serological testing 12 months after the end of treatment according to age group (full analysis set).

Treatment response	All patients		0 to 27 d		28 d to <8 m		8 m to <2 y		2 y to <18 y	
	NFX 60 d, n = 219	NFX 30 d, n = 111	NFX 60 d, n = 4	NFX 30 d, n = 3	NFX 60 d, n = 8	NFX 30 d, n = 4	NFX 60 d, n = 17	NFX 30 d, n = 8	NFX 60 d, n = 190	NFX 30 d, n = 96
Seroreduction, n	62	16	0	0	0	0	14	6	48	10
Negative seroconversion, n	10	5	3	3	7	2	0	0	0	0
Total, n	72	21	3	3	7	2	14	6	48	10
Rate (95% CI), %	32.9 (26.4, 39.3)	18.9 (11.2, 26.7)	75 (20.1, 100)	100 (83.3, 100)	87.5 (58.3, 100)	50 (0, 100)	82.4 (61.3, 100)	75 (38.7, 100)	25.3 (18.8, 31.7)	10.4 (3.8, 17.0)
Treatment difference (95% CI)	14.0 (3.7, 24.2)		25 (–96.6, 46.6)		37.5 (–35.3, 100)		7.4 (–36.9, 51.6)		14.9 (5.4, 24.3)	

d, days; m, months; y, years; CI, confidence interval.

Treatment difference refers to the comparison of treatment response rate for the 60-day and 30-day nifurtimox regimens with an asymptotic 2-sided 95% CI for the difference. The difference in efficacy between the two groups was confirmed if the 95% CI did not include 0.

Response was defined as seroreduction (in patients ≥8 months to <18 years of age at randomisation) or negative seroconversion (in all patients). Seroreduction was defined as a ≥20% reduction in optical density) measured by two conventional enzyme-linked immunosorbent assay (ELISA) serology tests, and negative seroconversion was defined as negative immunoglobulin G concentration measured by two conventional ELISA serology tests.

Patients for whom conventional serology results at 12-month follow-up were missing were considered as failures (i.e., non-responders).

### Secondary outcomes

ECG abnormality was the most frequently reported sign or symptom of Chagas disease. Bradycardia was noted in two patients in the 60-day treatment group at baseline (0.6% of the total) and three patients (one each with bradycardia, respiratory arrhythmia, and a slight deviation of the QRS axis) in each treatment group 12 months after treatment (1.9% of total). These findings were considered non-pathological by the investigator and not clinically significant. All other signs or symptoms, such as anaemia, hepatomegaly or Romana’s sign, were reported in no more than two of all patients (≤0.6%) at any study visit. Concentration tests for *T*. *cruzi* performed in infants aged <8 months, which were positive in all 19 patients at baseline, decreased over time and were negative in 18 patients (94.7% of total) at 30 days after the end of treatment; at this study visit, one patient in the 60-day treatment group was positive for the parasite by this test.

IHA results were negative at baseline for two patients in the 60-day treatment group. A decrease in mean IHA titre was observed in both nifurtimox treatment groups. At 12-month follow-up, 16 patients had negative IHA results: 11 patients (5.0%) after 60-day treatment and five (4.5%) after 30-day treatment (Table 4). Among the 10 patients with a negative seroconversion by conventional ELISA serology at 12-month follow-up in the 60-day treatment group, eight also showed negative serology measured by IHA and two had an IHA titre of 1/16, which is the lower titre limit considered sera reactive for anti-*T*. *cruzi* antibodies. All 10 patients had a negative parasitological concentration test for *T*. *cruzi* after the end of treatment. Negative qPCR results were also observed in all 10 patients consistently from Day 30 of the treatment phase until 12 months after treatment, confirming parasitological treatment response.

**Table 4:** Serological responses to 60-day and 30-day nifurtimox (NFX) treatment assessed 12 months after the end of treatment by indirect haemagglutination testing (full analysis set).

	Baseline			12 months after the end of treatment		
	NFX 60 d (n = 219)	NFX 30 d (n = 111)	Total (N = 330)	NFX 60 d (n = 219)	NFX 30 d (n = 111)	Total (N = 330)
Response, n (%)						
Reactive	216 (98.6)	111 (100)	327 (99.1)	199 (90.9)	103 (92.8)	302 (91.5)
Non-reactive	2 (0.9)	0	2 (0.6)	11 (5.0)	5 (4.5)	16 (4.8)
Missing	1 (0.5)	0	1 (0.3)	9 (4.1)	3 (2.7)	12 (3.6)
Titre*						
Mean (SD)	10.3 (2.11)	10.6 (1.99)	10.4 (2.07)	9.5 (2.35)	9.8 (2.33)	9.6 (2.35)
Median (range)	11 (4–12)	12 (5–12)	11 (4–12)	10 (4–12)	11 (4–12)	10 (4–12)

d, days; SD, standard deviation.

*Titres are given as–log_2_ transformed values.

The number of patients who were seronegative for the F-29 antigen increased from baseline over time. Interestingly, however, no F29 antigen could be detected at baseline in some patients. Most of these patients remained F29 antigen-negative at subsequent evaluations. To assess the effect of nifurtimox better, a sub-population was selected consisting of all patients with positive F-29 ELISA responses at baseline (n = 214, 64.8%). In this subpopulation, 66 patients (30.8%) had negative F-29 ELISA responses at 12-month follow-up (46 of 142 patients [32.4%] in the 60-day regimen and 20 of 72 patients [27.8%] in the 30-day regimen) (Table 5).

**Table 5:** Serological responses to 60-day and 30-day nifurtimox (NFX) treatment assessed 12 months after the end of treatment by non-conventional (F-29) ELISA (full analysis set [FAS] and sub-population of FAS).

Response, n (%)	Baseline			12 months after the end of treatment: all patients			12 months after the end of treatment: patients with positive F-29 results at baseline		
	NFX 60 d (n = 219)	NFX 30 d (n = 111)	Total (N = 330)	NFX 60 d (n = 219)	NFX 30 d (n = 111)	Total (N = 330)	NFX 60 d (n = 219)	NFX 30 d (n = 111)	Total (N = 330)
Reactive	142 (64.8)	72 (64.9)	214 (64.8)	96 (43.8)	54 (48.7)	150 (45.5)	94 (42.9)	51 (46.0)	145 (43.9)
Non-reactive	77 (35.2)	39 (35.1)	116 (35.2).	114 (52.1)	54 (48.7)	168 (50.9)	46 (21.0)	20 (18.0)	66 (20.0)
Missing	0	0	0	9 (4.1)	3 (2.7)	12 (3.6)	2 (0.9)	1 (0.9)	3 (0.9)

d, days.

The frequency of *T*. *cruzi* DNA detectable by qPCR decreased from 53.4% at baseline to 1.4% at the end of treatment and remained at this level through to the 12-month follow-up in the 60-day treatment group. Detectable qPCR results in the 30-day treatment group decreased from 51.4% at baseline to 4.5% at 12-month follow-up (S2 Table). Sequential qPCR analysis showed that the majority of patients with negative qPCR results at end of treatment remained negative at 12-month follow-up; detectable *T*. *cruzi* DNA was found in only eight patients (2.4%) at the 12-month post-treatment follow-up.

In total, 40/44 patients younger than 2 years (90.9%) and 134/286 older than 2 years (46.9%) tested positive by qPCR at baseline. Only four patients in each age group (2.4% of all patients) had positive qPCR results at 12-month follow-up. By country, positive qPCR results were found at baseline in 119/178 patients in Argentina, 45/62 patients in Bolivia and 10/90 patients in Colombia. Of the eight patients with positive qPCR results at 12-month follow-up post-treatment, five were from Argentina, two from Bolivia and one from Colombia.

### Safety

Nifurtimox was well tolerated. Treatment-emergent adverse events (TEAEs) were reported in 64.5% (213/330) of patients (S3 Table). Most TEAEs were mild or moderate in intensity and resolved without sequelae. The overall frequencies of TEAEs and study-drug-related TEAEs were slightly higher in the 60-day treatment group than the 30-day group; TEAEs in the 60-day treatment group were more frequent in those aged <12 years, and less frequent in newborns and those aged ≥12 years, than in the 30-day treatment group. There were no between-treatment differences in the frequency of serious TEAEs. TEAEs were less frequently observed in young children and babies than in adolescents (12–18 years of age) in both the 60-day and 30-day nifurtimox treatment groups. The most common TEAEs occurring in each age group are shown in S4 Table. Overall, 91 patients (27.6%) had study drug-related TEAEs but only 4.2% of any TEAEs led to discontinuation of study drug. No significant ECG abnormalities or abnormalities in laboratory parameters (haematology, blood chemistry and urinalysis) were observed during the treatment and follow-up periods in either treatment group.

## Discussion

The results of the current study confirmed the efficacy and safety of nifurtimox in paediatric patients with Chagas disease. This disease is one of five parasitic diseases targeted by the CDC for public health action based on the severity of the illness and the availability of effective treatment [10]. Treatment is now recommended for all infected children (aged <18 years) [10,12]. Nifurtimox is one of only two antitrypanosomal agents with proven efficacy that are approved in some Latin American countries for the treatment of Chagas disease [12].

Given the efficacy of nifurtimox anticipated from previous studies [15–17], clinical experience with the drug and the duration of follow-up needed to establish efficacy especially in chronic Chagas disease, it was not considered appropriate to conduct a prospective placebo-controlled study in children with Chagas disease [25,26]. Limitations of using such historical controls include potential differences between the patients in the present study and those in the historical control groups, in terms of age, time and setting of diagnosis and treatment. In this respect, the historical data relate to findings in 6- to 12-year-old Argentinian children only.

The primary efficacy analysis showed superiority of nifurtimox over historical placebo controls, with a treatment response rate of 32.9% with the 60-day nifurtimox regimen. Conversion of serological response to negative in the two conventional ELISA serology tests was observed in 10 patients at 12-month post-treatment follow-up, all of whom were younger than 8 months. This outcome was consistent with the results of IHA and qPCR tests. Treatment success is difficult to measure using the current criterion for cure–conversion of the anti-*T*. *cruzi* serological response to negative measured by conventional serological tests–because disease-specific antibodies persist for a long time even after successful antiparasitic treatment. In adult patients in the chronic phase of the disease, it can take 10 to 20 years after treatment before these patients become seronegative as assessed by conventional serological methods [8]. Although the duration of follow-up was limited to 12 months after treatment, the majority of the patients below the age of 8 months converted to seronegativity, confirming the efficacy of nifurtimox and emphasising the need to initiate the antiparasitic therapy as soon as possible after diagnosis of the infection.

In this study, the efficacy endpoint was based upon negative seroconversion or a target ≥20% decrease in optical density (seroreduction) measured by conventional ELISA serological tests. Because of the persistence of passively-transferred maternal anti-*T*. *cruzi* IgG in infants born to seropositive mothers during early life [27,28], we chose seroreduction as one of the component definitions of the primary outcome only in patients aged between 8 months and <18 years at randomisation. The threshold for seroreduction was based on the results of a large (n = 106) randomised clinical trial conducted in paediatric patients with Chagas disease, in which a 21% reduction in optical densities measured by conventional ELISA was observed in patients aged 6 to 12 years treated with 60-day benznidazole at 12-month follow-up [19]. Relying on consistent negative results from conventional serological tests means that it may take years to evaluate drug efficacy in older children with chronic Chagas disease, emphasising the need for improved markers of therapeutic response. A decrease in antibody titres with clearance of *T*. *cruzi* in blood seems to precede complete reversion of serological response to negative and may indicate an evolution towards cure from a clinical point of view [24]. In contrast, follow-up of patients in the chronic phase of Chagas disease has shown that antibody titres remain stable if no antitrypanosomal treatment is received [19,20]. Thus, seroreduction could be a useful predictor of future negative seroconversion; however, further follow-up assessments of the serological response are necessary to demonstrate its usefulness. The second phase of this study, CHICO SECURE, which is in progress, will contribute valuable information on the serological response during long-term follow-up of this patient cohort.

The serological treatment response rates achieved by the 60-day nifurtimox regimen in this study were compared with other studies evaluating antitrypanosomal drug treatment. In a placebo-controlled study that was one of the sources for the historical control values used in the current study [19], 5/53 patients (9.4%) aged 6–12 years treated with benznidazole 5 mg/kg/day for 60 days were seronegative by one conventional ELISA test at 12-month follow-up [19,29], compared with a negative seroconversion rate of 4.6% (10/219 patients) in the 60-day treatment group and of 4.5% (5/111 patients) in the 30-day treatment group by two conventional ELISAs in the current study. Similarly, in the historical control study, seropositivity for non-conventional F-29 ELISA decreased from 73% of children prior to benznidazole treatment to 55% after 12 months [29]. Successful clearance of the *T*. *cruzi* parasite following nifurtimox treatment in children has been reported previously [23]: negative seroconversion for both ELISA and indirect immunofluorescence was achieved in approximately 42% of children aged 4–19 years at 30 months after 60-day treatment with nifurtimox [23]. However, active vectorial transmission has been reported in the area where the latter study was conducted, so it is possible that reinfection resulted in a lower rate of negative seroconversion. Indeed, comparison of serological responses between different studies is confounded by numerous factors, including the target characteristic and performance of the test method employed, the parasite lineage and patient-related variables such as age when treated and length of follow-up [30]. For our investigation, we set up the first paediatric network for the clinical study of Chagas disease in Latin America. This network includes 25 centres located in Colombia (n = 4), Bolivia (n = 3) and Argentina (n = 18) in areas under active vector control.

Current efforts to improve therapy for Chagas disease focus on shorter treatment duration with consistent efficacy. The current study therefore also evaluated the efficacy of a nifurtimox treatment regimen of 30 days. Although efficacy of the shorter treatment was not comparable to the 60-day treatment regimen for the overall study population from a serological point of view, the serological and parasitological response rates of the two treatment regimens appeared to be similar in patients who were younger than 2 years at randomisation. This observation should be interpreted with caution, however, as the overall number of patients of this age was low and no formal statistical testing was performed.

The time to seronegative conversion appears to be inversely related to the duration of infection [9,31]. In newborn infants treated with benznidazole or nifurtimox, recovery was confirmed at 9 months in most, with *T*. *cruzi* antibody titre dropping to the detection cut-off after 9–15 months of life [32]. In children and adolescents, decreases in *T*. *cruzi* antibody titre may occur several years after treatment, requiring long-term follow-up, potentially for decades [4,23,33]. Follow-up studies also suggest that the earlier children are treated with antitrypanosomal drugs, the higher the rate of conversion from positive to negative serological results, highlighting the importance of early diagnosis and treatment of Chagas disease [4,9,24]. The need for such long-term follow-up of *T*. *cruzi* antibody titres with the use of conventional serology has led to the evaluation of alternative serological techniques as potential cure markers, with time to negative seroconversion occurring sooner than that observed with traditional methods. Of these methods, non-conventional F-29 ELISA, though not a definitive diagnostic test of Chagas disease, has emerged as a useful early indicator of cure in patients with chronic Chagas disease treated with antitrypanosomal drug, with disappearance of F-29 antigens significantly anticipating negative seroconversion when compared with conventional serology [19,33,34]. F-29 ELISA was used in the current study because the duration of post-treatment follow-up was limited to 12 months. The finding that approximately 31% of the subpopulation of patients with F-29 ELISA positivity at baseline showed negative F-29 ELISA responses at 12 months post-treatment is considered comparable to results previously reported for children aged 6 to 12 years treated with benznidazole [19,29].

In recent years, the availability of PCR to detect *T*. *cruzi* DNA in blood samples has provided new possibilities for the diagnosis and evaluation of response to trypanocidal chemotherapy, allowing parasitic load to be quantified in samples from patients with *T*. *cruzi* infection [35]. In the current study, parasite DNA was detected in 53% of single samples from patients at baseline. Clearance of parasitaemia measured by qPCR was demonstrated in the majority of nifurtimox-treated patients. A positive qPCR result, which is considered indicative of treatment failure [24], was observed in only 1.4% of patients in the 60-day regimen and 4.5% in the 30-day regimen at 12-month follow-up. More than 90% of patients with detectable *T*. *cruzi* DNA at baseline had undetectable results at 12-month follow-up, indicating the good parasiticidal effects of nifurtimox. PCR was negative in over 95% of patients at the end of treatment and remained negative in most of these patients during long-term follow-up. Positive serology in patients converting to *T*. *cruzi* qPCR-negative after treatment could be explained by immune reactivity continuing in response to host or other persistent or recurrent antigens in otherwise cured patients [24]. A study of therapeutic response to benznidazole 5–8 mg per kg/day for 60 days in 107 children (average age at baseline, 6.9 years) determined a failure rate of 2.6% by positive PCR at 12 months of follow-up [24]. In contrast, to these findings and those of the current study in children, it has been reported that 18% of adults with chronic Chagas disease experienced therapeutic failure (evaluated by qPCR) 12 months after receiving treatment with benznidazole 5 mg/kg/day for 60 days, reinforcing the importance of early identification and treatment of the disease [36].

Nifurtimox was well tolerated by the paediatric patients in this study. Less than 30% of TEAEs were assessed as being related to nifurtimox treatment and only 4.2% resulted in treatment discontinuation. When compared with a commonly accepted definition [37], overall compliance was good; 87% of all patients took >80% to 120% of their assigned study medication and only 11% of patient could be considered poorly compliant. The TEAEs experienced were primarily mild to moderate in severity, resolved upon treatment cessation, and common TEAEs were consistent with previous nifurtimox studies [16,23]. In the current study, nifurtimox appeared to be better tolerated in young children and babies than in children older than 12 years.

Paediatric formulations of effective drugs used to treat Chagas disease are urgently needed in order to improve dosing accuracy in children and to lower the risk of over- or under-dosing. The current study found that nifurtimox administered as age- and weight-adjusted doses for 60 days in a large cohort of children achieved a superior serological response compared with placebo in historical controls and was well tolerated. Overall, the 60-day treatment regimen was more effective than the same dosing for 30 days. The 60-day and 30-day nifurtimox regimens appeared to be equally efficacious in children younger than 2 years; however, based on the low number of patients in these age groups and the lack of formal statistical testing, the results should be interpreted with caution. Overall, our findings, despite acknowledged limitations, support current recommendations to reduce the time from infection to treatment, thereby increasing the likelihood of an objective therapeutic response and seroconversion. Further long-term follow-up of patients in this study will provide important information about the progress of serological conversion in children treated with nifurtimox, as well as the potential efficacy difference between the two regimens over time.

## Supporting information

S1 Text CHICO Study Group study site principal investigators. (DOCX)

S2 Text Exclusion criteria. (DOCX)

S1 Table Treatment duration and compliance. (DOCX)

S2 Table Responses to 60-day and 30-day nifurtimox (NFX) treatment assessed by quantitative polymerase chain reaction tests through treatment and follow-up (full analysis set). (DOCX)

S3 Table Summary of treatment-emergent adverse events (TEAE), study drug-related TEAEs and serious TEAEs (full analysis set). (DOCX)

S4 Table Summary of treatment-emergent adverse events (TEAE) by primary system organ class and preferred term according to age group (full analysis set). (DOCX)
