# Supplementary material for: Prospective, historically controlled study to evaluate the efficacy and safety of a new paediatric formulation of nifurtimox in children aged 0 to 17 years with Chagas disease one year after treatment (CHICO)
Source: PLoS Negl Trop Dis. 2021 Jan 7;15(1):e0008912. doi: 10.1371/journal.pntd.0008912 (PMC7790535; doi:10.1371/journal.pntd.0008912)
Supplement: S2 Text — (DOCX) [file pntd.0008912.s002.docx]

**S2 Text** Exclusion criteria

| Patients aged 0–27 days who were born pre-term (i.e. gestational age <37 weeks) or low birth weight (<2500 g) newborns and newborns with clinical signs of neonatal depression (maximum Apgar score <7 at 5 minutes) |
| --- |
| Patients with known evidence of conditions associated with Chagas disease, such as:   - heart disease or cardiomyopathy - gastrointestinal dysfunction or digestive disease - serious manifestations of acute Chagas disease, including myocarditis, meningoencephalitis, or pneumonitis - peripheral nervous system damage or peripheral neuropathy - clinically significant psychiatric disorder or epilepsy |
| Patients with contraindications or warnings to nifurtimox administration, or with conditions that might increase the risk of the undesirable effects of nifurtimox, including:   - hypersensitivity to nifurtimox or other hydantoin, or to any of the medication excipients - suspected or known porphyria - severe renal impairment, defined as   - for patients younger than 1 year, estimated glomerular filtration rate (eGFR) <100% of the lower limit of normal (LLN) appropriate for age   - for patients aged ≥1 year to <18 years, eGFR <80% of LLN appropriate for age - severe hepatic impairment, as evidenced by alanine aminotransferase or aspartate aminotransferase increased to levels considered to be clinically significant by the investigator - history of brain injury, predisposition to seizures or epilepsy, psychiatric disease, or serious behavioral alteration - severe or significant gastrointestinal disorders, or metabolism and nutrition disorders |
| Patients with a history of malignancy in the previous 5 years |
| Patients who are chronic abusers or current users of alcohol or recreational drugs, or who are newborn of mothers who are chronic abusers or current users of alcohol or recreational drugs |
| Patients with any condition that would prevent him or her taking oral medication |
| Immunocompromised patients |
| Patients with any other acute or chronic health condition or congenital disorder that, in the opinion of the investigator, would make them unsuitable for participation in a clinical study, or that may interfere with the efficacy, safety and/or pharmacokinetic evaluations of the study drug |
| Patients who have had previous treatment with trypanocidal agents or an accepted indication for antiparasitic therapy |
| Patients who are pregnant or breastfeeding |
| Offspring of breastfeeding mothers receiving treatment with trypanocidal agents |
| Patients who the investigator considers unlikely to adhere to adhere to the clinical study protocol, comply with study drug administration, or complete the study and follow-up |
| Patients with close affiliation with the investigational site |
| Residential conditions where there was no active or effective vector control to *T. cruzi* reinfection as determined by the respective health authority guidelines |
