# Supplementary material for: Prospective, historically controlled study to evaluate the efficacy and safety of a new paediatric formulation of nifurtimox in children aged 0 to 17 years with Chagas disease one year after treatment (CHICO)
Source: PLoS Negl Trop Dis. 2021 Jan 7;15(1):e0008912. doi: 10.1371/journal.pntd.0008912 (PMC7790535; doi:10.1371/journal.pntd.0008912)
Supplement: S1 Table — (DOCX) [file pntd.0008912.s003.docx]

**S1 Table.** Treatment duration and compliance.

|  | **Nifurtimox 60-day regimen (n=219)** | **Nifurtimox 30-day regimen (n=111)** | |
| --- | --- | --- | --- |
|  |  | **Nifurtimox 30 days** | **Placebo 30 days** |
| Treatment duration, n (%) |  |  |  |
| Confirmed | 219 (100) | 110 (99.1) | 106 (95.5) |
| Missing | 0 | 1 (0.9) | 5 (4.5) |
| Duration, days |  |  |  |
| Mean (SD) | 58.9 (9.1); | 29.4 (3.6); | 31.2 (3.0); |
| Median (range) | 61 (10–85) | 29 (10–40) | 31 (17–40) |
| Compliance categories, n (%) |  |  |  |
| Confirmed | 218 (99.5) | 110 (99.1) | 106 (95.5) |
| Missing | 1 (0.5) | 1 (0.9) | 5 (4.5) |
| ≤80% | 24 (11.0) | 14 (12.6) | 10 (9.0) |
| >80% to 120% | 193 (88.1) | 94 (84.7) | 95 (85.6) |
| >120% | 1 (0.5) | 2 (1.8) | 1 (0.9) |
| Compliance, mean (SD) %* | 92.3 (12.8) | 92.9 (14.0) | 91.3 (12.9) |

SD, standard deviation. *Compliance % was defined as the number of tablets taken divided by the number of tablets recommended by study protocol x 100.
