# Supplementary material for: Prospective, historically controlled study to evaluate the efficacy and safety of a new paediatric formulation of nifurtimox in children aged 0 to 17 years with Chagas disease one year after treatment (CHICO)
Source: PLoS Negl Trop Dis. 2021 Jan 7;15(1):e0008912. doi: 10.1371/journal.pntd.0008912 (PMC7790535; doi:10.1371/journal.pntd.0008912)
Supplement: S2 Table — (DOCX) [file pntd.0008912.s004.docx]

**S2 Table.** Responses to 60-day and 30-day nifurtimox (NFX) treatment assessed by quantitative polymerase chain reaction tests through treatment and follow-up (full analysis set).

| **Study visit: timepoint** | **Result** | Nifurtimox 60-day regimen (n=219) | **Nifurtimox 30-day regimen (n=111)** | **Total (N=330)** |
| --- | --- | --- | --- | --- |
| Visit 1: day −14 to −1 | Non-detectable | 99 (45.2) | 53 (47.8) | 152 (46.1) |
|  | Detectable | 117 (53.4) | 57 (51.4) | 174 (52.7) |
|  | Non-evaluable | 1 (0.5) | 1 (0.9) | 2 (0.6) |
|  | Missing | 2 (0.9) | 0 | 2 (0.6) |
|  | n | 219 (100) | 111 (100) | 330 (100) |
| Visit 3: day 7 ± 1 | Non-detectable | 171 (78.1) | 86 (77.5) | 257 (77.9) |
|  | Detectable | 46 (21.0) | 21 (18.9) | 67 (20.3) |
|  | Non-evaluable | 0 | 1 (0.9) | 1 (0.3) |
|  | Missing | 2 (0.9) | 3 (2.7) | 5 (1.5) |
|  | n | 219 (100) | 111 (100) | 330 (100) |
| Visit 6: day 30 ± 3 | Non-detectable | 207 (94.5) | 105 (94.6) | 312 (94.6) |
|  | Detectable | 4 (1.8) | 3 (2.7) | 7 (2.1) |
|  | Non-evaluable | 3 (1.4) | 2 (1.8) | 5 (1.5) |
|  | Missing | 5 (2.3) | 1 (0.9) | 6 (1.8) |
|  | n | 219 (100) | 111 (100) | 330 (100) |
| Visit 8 (EOT): day 60 ± 3 | Non-detectable | 210 (95.9) | 105 (94.6) | 315 (95.5) |
|  | Detectable | 3 (1.4) | 1 (0.9) | 4 (1.2) |
|  | Non-evaluable | 2 (0.9) | 2 (1.8) | 4 (1.2) |
|  | Missing | 4 (1.8) | 3 (2.7) | 7 (2.1) |
|  | n | 219 (100) | 111 (100) | 330 (100) |
| Visit 10: day 240 ± 7 | Non-detectable | 206 (94.1) | 105 (94.6) | 311 (94.2) |
|  | Detectable | 3 (1.4) | 2 (1.8) | 5 (1.5) |
|  | Non-evaluable | 2 (0.9) | 2 (1.8) | 4 (1.2) |
|  | Missing | 8 (3.7) | 2 (1.8) | 10 (3.0) |
|  | n | 219 (100) | 111 (100) | 330 (100) |
| Visit 11: day 420 ± 7 | Non-detectable | 205 (93.6) | 102 (91.9) | 307 (93.0) |
|  | Detectable | 3 (1.4) | 5 (4.5) | 8 (2.4) |
|  | Non-evaluable | 1 (0.5) | 1 (0.9) | 2 (0.6) |
|  | Missing | 10 (4.6) | 3 (2.7) | 13 (3.9) |
|  | n | 219 (100) | 111 (100) | 330 (100) |

EOT, end of treatment
