# Supplementary material for: Prospective, historically controlled study to evaluate the efficacy and safety of a new paediatric formulation of nifurtimox in children aged 0 to 17 years with Chagas disease one year after treatment (CHICO)
Source: PLoS Negl Trop Dis. 2021 Jan 7;15(1):e0008912. doi: 10.1371/journal.pntd.0008912 (PMC7790535; doi:10.1371/journal.pntd.0008912)
Supplement: S3 Table — (DOCX) [file pntd.0008912.s005.docx]

**S3 Table.** Summary of treatment-emergent adverse events (TEAE), study drug-related TEAEs and serious TEAEs (full analysis set).

|  | **Nifurtimox 60-day regimen (n=219)** | **Nifurtimox 30-day regimen (n=111)** | **Total (N=330)** |
| --- | --- | --- | --- |
| Any TEAE | 147 (67.1) | 66 (59.5) | 213 (64.5) |
| 0 to 27 d | 3 (1.4) | 2 (1.8) | 5 (1.5) |
| 28 d to <2 y | 19 (8.7) | 6 (5.4) | 25 (7.6) |
| 2 y to <6 y | 18 (8.2) | 5 (4.5) | 23 (7.0) |
| 6 y to <12 y | 48 (21.9) | 15 (13.5) | 63 (19.1) |
| 12 y to <18 y | 59 (26.9) | 38 (34.2) | 97 (29.4) |
| Maximum intensity for any TEAE | | | |
| Mild | 108 (49.3) | 55 (49.5) | 163 (49.4) |
| Moderate | 37 (16.9) | 10 (9.0) | 47 (14.2) |
| Severe | 2 (0.9) | 1 (0.9) | 3 (0.9) |
| Any TEAE related to procedures required by the study protocol | 0 | 0 | 0 |
| Any TEAE leading to treatment discontinuation | 12 (5.5) | 2 (1.8) | 14 (4.2) |
| Any study-drug-related TEAE | 62 (28.3) | 29 (26.1) | 91 (27.6) |
| Maximum intensity for any study-drug-related TEAE | | | |
| Mild | 41 (18.7) | 23 (20.7) | 64 (19.4) |
| Moderate | 20 (9.1) | 6 (5.4) | 26 (7.9) |
| Severe | 1 (0.5) | 0 | 1 (0.3) |
| Any serious TEAE | 6 (2.7) | 3 (2.7) | 9 (2.7) |
| Any study-drug-related serious TEAE | 2 (0.9) | 1 (0.9) | 3 (0.9) |
| Any serious TEAE related to procedures required by the study protocol | 0 | 0 | 0 |
| Any serious TEAE leading to treatment discontinuation | 2 (0.9) | 1 (0.9) | 3 (0.9) |
| Any TEAE resulting in patient death | 0 | 0 | 0 |
| Common TEAEs (≥5% of all patients) | | | |
| Headache | 28 (12.8) | 16 (14.4) | 44 (13.3) |
| Vomiting | 32 (14.6) | 9 (8.1) | 41 (12.4) |
| Nausea | 18 (8.2) | 14 (12.6) | 32 (9.7) |
| Decreased appetite | 23 (10.5) | 8 (7.2) | 31 (9.4) |
| Abdominal pain | 15 (6.8) | 8 (7.2) | 23 (7.0) |
| Nasopharyngitis | 14 (6.4) | 6 (5.4) | 20 (6.1) |
| Pyrexia | 16 (7.3) | 3 (2.7) | 19 (5.8) |
| Upper abdominal pain | 14 (6.4) | 4 (3.6) | 18 (5.5) |

d, days, y, years.

Data shown are n (%).

Events were considered treatment-emergent if they occurred any time after the first application of study drug during the course of the study, up to and including 7 days after the last application of study drug.
