# Supplementary material for: Prospective, historically controlled study to evaluate the efficacy and safety of a new paediatric formulation of nifurtimox in children aged 0 to 17 years with Chagas disease one year after treatment (CHICO)
Source: PLoS Negl Trop Dis. 2021 Jan 7;15(1):e0008912. doi: 10.1371/journal.pntd.0008912 (PMC7790535; doi:10.1371/journal.pntd.0008912)
Supplement: S4 Table — (DOCX) [file pntd.0008912.s006.docx]

**S4 Table.** Summary of treatment-emergent adverse events (TEAE) by primary system organ class and preferred term according to age group (full analysis set).

| **Primary system organ class** | **0 to 27 d** | **28 d to <2 y** | **2 y to <6 y** | **6 y to <12 y** | **12 y to <18 y** |
| --- | --- | --- | --- | --- | --- |
| Patients with at least one TEAE | 5 (1.5) | 25 (7.6) | 23 (7.0) | 63 (19.1) | 97 (29.4) |
| Blood and lymphatic system disorders | 3 (0.9) | 4 (1.2) | 4 (1.2) | 7 (2.1) | 3 (0.9) |
| Ear and labyrinth disorders | 0 | 0 | 0 | 0 | 1 (0.3) |
| Eye disorders | 0 | 0 | 0 | 1 (0.3) | 1 (0.3) |
| Gastrointestinal disorders | 2 (0.6) | 16 (4.8) | 11 (3.3) | 29 (8.8) | 53 (16.1) |
| General disorders and administration site conditions | 0 | 6 (1.8) | 3 (0.9) | 8 (2.4) | 13 (3.9) |
| Hepatobiliary disorders | 0 | 0 | 0 | 1 (0.3) | 2 (0.6) |
| Immune system disorders | 0 | 0 | 1 (0.3) | 0 | 0 |
| Infections and infestations | 2 (0.6) | 12 (3.6) | 13 (3.9) | 28 (8.5) | 26 (7.9) |
| Injury, poisoning, and procedural complications | 0 | 0 | 0 | 2 (0.6) | 4 (1.2) |
| Investigations | 0 | 0 | 2 (0.6) | 3 (0.9) | 5 (1.5) |
| Metabolism and nutrition disorders | 0 | 1 (0.3) | 6 (1.8) | 8 (2.4) | 18 (5.5) |
| Musculoskeletal and connective tissue disorders | 0 | 0 | 0 | 1 (0.3) | 6 (1.8) |
| Nervous system disorders | 0 | 1 (0.3) | 2 (0.6) | 17 (5.2) | 33 (10.0) |
| Psychiatric disorders | 0 | 1 (0.3) | 1 (0.3) | 0 | 1 (0.3) |
| Renal and urinary disorders | 0 | 0 | 0 | 1 (0.3) | 2 (0.6) |
| Reproductive system and breast disorders | 0 | 0 | 1 (0.3) | 0 | 0 |
| Respiratory, thoracic, and mediastinal disorders | 1 (0.3) | 5 (1.5) | 3 (0.9) | 4 (1.2) | 13 (3.9) |
| Skin and subcutaneous tissue disorders | 0 | 3 (0.9) | 2 (0.6) | 9 (2.7) | 14 (4.2) |

d, days; y, years.

Data shown are n (%) calculated from the full analysis set (n=330).
